# Variation in the *TAS2R38* Bitterness Receptor Gene Was Associated with Food Consumption and Obesity Risk in Koreans

**DOI:** 10.3390/nu11091973

**Published:** 2019-08-21

**Authors:** Jeong-Hwa Choi

**Affiliations:** Department of Food Science and Nutrition, Keimyung University, 1095 Dalgubeol-daero, Dalseo-Gu, Daegu 42601, Korea; jhchoi@kmu.ac.kr; Tel.: +82-53-580-5913

**Keywords:** bitter taste, body mass index, dietary intake, Korean obesity, *TAS2R38*

## Abstract

Bitterness-sensing protein taste receptor type-2 member 38 (*TAS2R38*, T2R38) mediates taste perception and various physiological responses, including energy- and adiposity-related mechanisms. This study examined whether the genetic variant rs10246939 C > T in *TAS2R38* was associated with food intake and body size as well as obesity risk. Data from the Korean Multi-Rural Communities Cohort study (1338 males and 2229 females) were analyzed to obtain the intake of six food groups, alcohol consumption, smoking status and anthropometric measurements, including height, weight, waist and hip circumference, and body mass index (BMI), according to the rs10246939 genotype. Findings suggested that females with the TT genotype consumed more fruit (adjusted *p* = 0.025) and had significantly higher body weights (adjusted *p* = 0.046) and BMIs (adjusted *p* = 0.003) than individuals with other genotypes. Having the TT genotype also increased the risk of obesity by 1.75-fold (95% confidence interval: 1.31–2.36) in females. The genetic variation had a minimal influence on the males’ dietary intake, but tended to increase the adiposity risk. In conclusion, *TAS2R38* rs10246939 variation was associated with Koreans’ dietary intake and increased their risk of obesity. Although more detailed statistical analyses in the larger cohort are required, current study suggested that, as a genetic predictive marker, *TAS2R38* bitterness receptor variations may have a large implication in obesity prevention and treatment.

## 1. Introduction

Obesity occurs as a consequence of an imbalance between energy consumption and expenditure. An energy intake higher than the physical requirement results in the accumulation of excessive body fat, which may lead to chronic inflammatory responses and other health issues, including diabetes, hypertension, hyperlipidemia, atherosclerotic diseases and possibly cancer [1]. Therefore, to prevent obesity, studies have been conducted to identify the risk factors related to physical activity, dietary behaviors and molecular etiology. For a more comprehensive approach, recent obesity studies have explored the common genetic variations involved in dietary intake, energy metabolism and adipogenesis regulation [2]. In line with this approach, polymorphisms in *PPARG2* and *IL6* have been identified as potential genetic markers for food consumption as well as body weight regulation [3,4]. However, little is known about genetic loci in taste sensing, despite its importance in dietary behavior and physiological metabolism in the context of obesity etiology.

Bitterness is known to be a major sensory element in the formation of preference and rejection of food and hence may regulate dietary intake [5]. In human bitterness perception, orally expressed bitterness receptors (taste receptor type 2, *TAS2Rs*, T2Rs) act as a signaling gateway [6]. Among the 25 isoforms of the *TAS2Rs* genes, *TAS2R38* and its encoded protein T2R38 are the most intensively studied factors in bitterness-sensing genetics. Studies have suggested that the diplotype of three genetic variations in *TAS2R38*, A49P (rs713598, G > C), V262A (rs1726866, T > C) and I296V (rs10246939, T > C), control the activity and expression of the receptor, thereby modifying bitterness sensitivity [6]. Individuals with the PAV haplotype (super taster) are more sensitive to the bitterness of phenylthiocarbamide (PTC) and 6-n-propylthiouracil (PROP); however, those with the AVI haplotype were less sensitive to those compounds (non-taster) [6]. Therefore, the *TAS2R38* diplotype was associated with differential intake of cruciferous vegetables, which contain glucosinolates with the thiourea moiety, an agonist of T2R38 [7]. Furthermore, the genetic variation influenced the intake of fruit, sweets, fat and alcohol over bitter-tasting foods [8,9,10,11].

T2R bitterness receptors are also expressed in extra-cavity locations, including the brain, respiratory tract, pancreas, gastrointestinal mucosa and thyroid [12]. As chemosensing G protein-coupled receptors, the extraoral expression of bitterness receptors plays a decisive role in the regulation of physiological metabolism and disease etiology [13,14,15]. The sensing of exterior/interior substances by bitterness receptors initiates the subsequent responses to process the stimuli. Therefore, the genetic variations in T2Rs possibly regulate the susceptibility to disease and physiological responses, independent of dietary intake. For instance, thyrocyte-expressed T2Rs regulate the production of triiodothyronine and thyroxin and are associated with the risk of papillary thyroid carcinoma [16,17]. The genetic variations in *TAS2R38* also modified the risk of gastric and colorectal cancer [13,14,18]. Observational studies reported that those genetic variations were associated with body fatness. Bitterness phenotype or *TAS2R38* genetic variations have been associated with weight, body mass index (BMI), and obesity [15,19,20]. However, the findings are still inconclusive: The influence of bitterness receptor genetic variations on BMI was not evident in some studies [9,21] or showed a sex-dimorphic association [20,22]. Epidemiological studies regarding *TAS2R38* genetic variations in Koreans have mainly focused on the risk of cancer incidence and dietary intake [13,14]. Given the wide implication of bitterness receptors in human health, *TAS2R38* genetic variations may be potential genetic markers for adiposity. However, evidence from a large-scale Korean population is lacking.

This study aimed to investigate the association between a *TAS2R38* genetic variation and dietary intake and body size in Koreans. The risk of obesity associated with the genetic variation was also estimated. The study analyzed data from the Korean Multi-Rural Communities Cohort study (MRCohort), a representative epidemiological study cohort of Koreans. As a genetic marker, *TAS2R38* rs10246939 T > C was applied. Individuals with the C allele are known to be sensitive to bitterness (taster, Val of PAV haplotype), while individuals with the T allele are associated with lower bitterness intensity (non-taster, Ile of AVI haplotype) [6].

## 2. Materials and Methods

### 2.1. Data Collection

**Figure 1:**
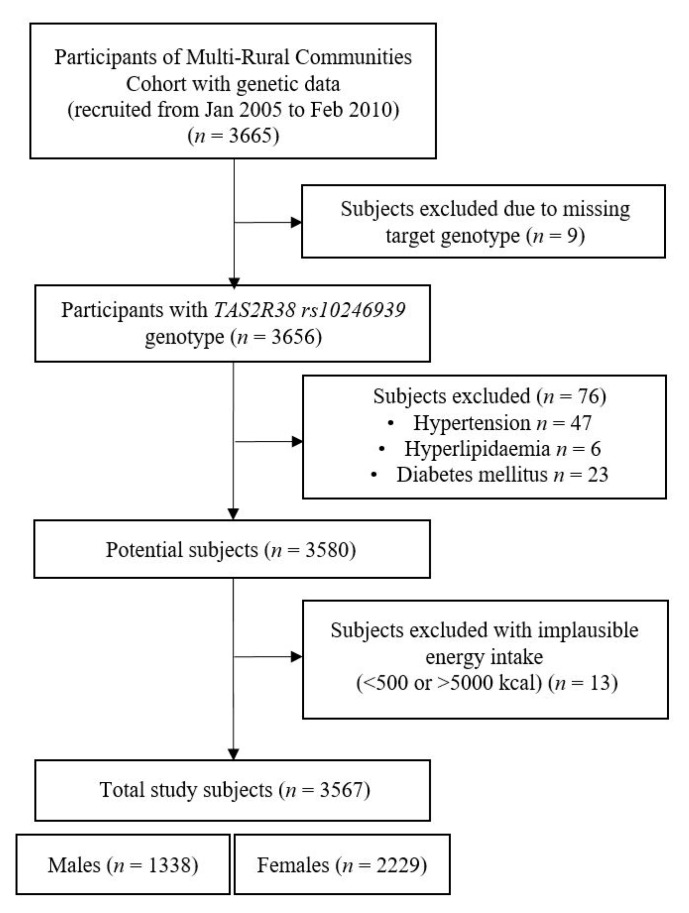
Simplified process of subject selection in the present study.

The present study was conducted with data from the MRCohort, a section of The Korean Genome Epidemiology Study (KoGES). The details of the MRCohort were mentioned previously elsewhere [23,24]. Briefly, subjects were recruited from three rural regions (Goryeong, Namwon, and Yangpyeong) between January 2005 and February 2010. Using the multistage cluster technique, certain villages in those areas were selected, and participants were mainly farmers and housewives. From the MRCohort dataset with genotype (*n* = 3,665), the subjects meeting the following criteria were excluded in this study: missing genetic data for target locus (*n* = 9); and physician-diagnosed hypertension (*n* = 47), hyperlipidemia (*n* = 6) or diabetes mellitus (*n* = 23). Additionally, subjects with implausible total energy intake (<500 or >5000 kcal/day) were also excluded (*n* = 13). Therefore, a total of 3567 participants (1338 males and 2229 females) aged from 40 to 89 years were analyzed in the study (Figure 1). The MRCohort protocol was approved by three Institutional Review Boards (IRBs; Hanyang University, Chonnam National University and Keimyung University). All participants provided written informed consent prior to the commencement of the study. Ethical approval for this study was also obtained after evaluation by the IRB (40525-201802-HR-121-01).

### 2.2. General Characteristics of the Study Subjects

Trained interviewers used a structured questionnaire to obtain the subjects’ descriptive and lifestyle characteristic data, including age, sex, tobacco use, alcohol consumption, education and the regular practice of exercise. Smoking and alcohol consumption status were defined as never, past or current. The level of education was defined as elementary graduate (≤6 years), high school graduate (7~12 years) and college level education or higher (>12 years). Regular exercise was defined as exercising ≥3 times per week for ≥30 min per session [24].

### 2.3. Dietary Intake Analyses

Dietary data were collected by trained interviewers using a validated food frequency questionnaire (FFQ) with 106 food items commonly consumed in Korea. Participants were asked to mark the frequency of consumption and the average serving size of the presented foods during the last year on the questionnaire. Nine frequencies (never or rarely, once a month, two or three times a month, once or twice a week, three or four times a week, five or six times a week, once a day, twice a day, or three times a day) and three serving sizes (small, medium, or large) for each food were described on the form. To answer whether the *TAS2R38* rs10246939 genetic variation may influence Koreans’ food intake, the consumption of the following six types of foods was investigated with a particular interest: total vegetables, cruciferous vegetables, dark green vegetables, fruits, citrus fruits and sweets (see Supplementary Table S1 for the food items in each group). The total daily alcohol intake (g/day) was calculated for current and past drinkers from commonly consumed types of alcoholic drinks, frequency of alcohol consumption, size of glass and the percentage of alcohol. Nutritional intake, including total energy and dietary fiber intake, was assessed using the Korean Food Composition Table (Seventh edition).

### 2.4. Anthropometric Measurements

Height and waist and hip circumference were determined on a 0.1 cm scale, and weight was estimated on a 0.01 kg scale with the participant in light clothing with no shoes. The ideal body weight was computed using the individual’s height with Broca’s method. BMI was calculated by dividing the weight (kg) by height squared (m^2^). Overweight and obesity were classified using BMI following clinical guidelines of the Korean Society for the Study of Obesity (2014) [25]. A BMI < 18.5 was classified as underweight, 18.5 ≤ BMI < 23 was normal, 23 ≤ BMI < 24.9 indicated overweight, and a BMI ≥ 25 indicated obesity.

### 2.5. Genotyping

Genomic DNA (gDNA) was isolated from peripheral blood drawn from participants using a commercial extraction kit (Intron Biotechnology, Gyeonggi-do, Korea). Illumina Omni 1 Quad bead microarrays with 748,585 loci of single nucleotide polymorphisms (Illumina Inc., San Diego, CA, USA) were used to genotype the isolated gDNA samples. The call rate for *TAS2R38* rs10246939 was above 95%, and the genetic variant was in the Hardy–Weinberg equilibrium [23,26,27].

### 2.6. Statistical Analyses

The differences in descriptive characteristics between *TAS2R38* rs10246939 genotypes were ascertained based on an analysis of variance (ANOVA) and chi-squared test. Dietary intake data were primarily adjusted for total energy intake with the residual method prior to the analyses [28]. The comparisons of dietary intake and anthropometric data between groups with different genotypes were performed using ANOVA, either with presence or absence of adjustments. *Post-hoc* tests were performed following Tukey’s methods. The differential distribution of adiposity grade by genotype was assessed by chi-squared tests. Lastly, the likelihood of obesity according to the genetic variant was determined using logistic regression models, either with the presence of absence of adjustments, and were presented as odds ratios (ORs) and 95% confidence intervals (95% CI). All statistical analyses were conducted using SAS version 9.3 (SAS Institute Inc., Cary, NC, USA). A two-tailed *p*-value < 0.05 was considered statistically significant.

## 3. Results

### 3.1. Distribution of the TAS2R38 Genotypes and Descriptive Data

The distribution of *TAS2R38* rs10246939 and the general characteristics of the study population are presented in Table 1. In males, 36.6, 48.2 and 15.3% of subjects had the CC, CT and TT genotypes, respectively, and in females, 33.4, 48.5 and 18.1% of the subjects possessed the CC, CT and TT genotypes, respectively. This distribution of the rs10246939 genotypes was similar to that reported from a previous Korean study (36.4, 46.0 and 17.5% for the CC, CT and TT genotypes, respectively) [14]. The general characteristics of the study subjects are also described in Table 1, and they take into account sex and *TAS2R38* rs10246939 genotype. In males and females, the genotype was not associated with significant differences in age, alcohol consumption behavior, education or the regular practice of exercise. Only females’ tobacco use differed among the genotypes (*p* = 0.041). These results suggest that age, alcohol consumption behavior, education and regular physical exercise, but not tobacco use, are independent of the *TAS2R38* rs10246939 genetic variant in this study population.

### 3.2. Dietary Intake, Alcohol Consumption and TAS2R38 Genotype

Table 2 shows the dietary intake and alcohol consumption levels for each *TAS2R38* rs10246939 genotype group in males and females. Males’ vegetable, cruciferous vegetable, dark green vegetable, fruit, citrus fruit, fiber, sweets and alcohol intake were not different between each genotype group, whether or not the subjects’ demographic and lifestyle factors were taken into account in the statistical models. The *TAS2R38* rs10246939 genotype group had a different total energy intake when only the covariates were adjusted (*p* = 0.024). However, in males, the genotype was not associated with a different percent of energy from carbohydrates, protein and fat. In contrast, the *TAS2R38* rs10246939 genetic variation showed a decisive influence on females’ dietary intake. Females with the TT genotype had higher total fruit intake (184.3 ± 7.46 g/day) than females with the CC and CT genotypes (*p* = 0.031; 163.6 ± 5.44 and 169.8 ± 4.48 g/day, respectively). This differential fruit intake for females with the TT genotype remained in the adjusted statistical model (*p* = 0.025). Additionally, females with the TT genotype also tended to intake more sweets (29.5 ± 2.74 g/day) compared to those with the CC or CT genotype (24.1 ± 1.74 and 26.5 ± 1.34 g/day, respectively), although the difference was not statistically significant. The *TAS2R38* rs10246939 genotype, however, showed no significant association with females’ intakes of the other investigated foods, total energy intake or percent of energy from macronutrients.

### 3.3. Anthropometric Data and TAS2R38 Genotype

The association between *TAS2R38* rs10246939 and body size is presented in Table 3. The genotype did not show a significant association with the male subjects’ measured body sizes. In contrast, the *TAS2R38* rs10246939 genetic variation had distinctive associations with weight, hip circumference and BMI in the female subjects. Females with the TT genotype had higher body weights (57.3 ± 0.43 kg) than those with the CC and CT genotypes (55.9 ± 0.3 and 56.0 ± 0.26 kg, crude *p* = 0.019 and adjusted *p* = 0.046, respectively). A similar and statistically significant trend of hip circumference and BMI was also evident: Female subjects with the TT genotype had a larger hip circumference (93.4 ± 0.33 cm) and BMI (24.5 ± 0.16) than those with the other two genotypes (for hip circumference, crude *p* = 0.048 and adjusted *p* = 0.045, for BMI, crude *p* = 0.002 and adjusted *p* = 0.003). However, the females’ other measured markers, including height, waist circumference, and ideal body weight showed minimal associations with the genotype.

### 3.4. Association between Adiposity and TAS2R38 Genotype

To ascertain whether the *TAS2R38* rs10246939 genetic variation is associated with adiposity in Koreans, the subjects were first grouped based on their obesity classification, genotype, and sex (Table 4). Chi-squared tests revealed that subjects’ obesity status was differentially distributed based on the rs10246939 genotype in females (*p* = 0.005), but not in males.

Lastly, the observed association between the *TAS2R38* rs10246939 genotype and adiposity is presented with ORs and 95% CIs in Table 5. The genetic variation was associated with the risk of overweight neither in males nor females. However, *TAS2R38* rs10246939 was a significant obesity risk-modifying factor in the female subjects. Females with the *TAS2R38* rs10246939 variation showed an increased risk of obesity based on the number of T alleles. Females with the CT or TT genotypes were more likely to be obese than those with the CC reference genotype, with approximately 1.29- and 1.74-fold increased risks, respectively. Such obesity risk modified by the *TAS2R38* genetic variation was also maintained in the adjusted models: The OR for the CT genotype was 1.33 (95% CI: 1.06–1.67) and for the TT genotype was 1.75 (95% CI: 1.31–2.36). The similar trend toward was found regarding *TAS2R38* rs10246939 T allele and the increased risk of obesity in males, but the statistical power was lacked (adjusted OR for TT genotype: 1.45, 95%CI: 0.95-2.22, *p* < 0.081). Finally, the risk of obesity was estimated in entire cohort. The adjusted statistical models predicted that having of TT genotype increased the risk of obesity 1.61 times, compared to reference CC genotype (95% CI: 1.27–2.04).

## 4. Discussion

Taste perception is a decisive factor in the formation of an individual’s dietary behavior and is also involved in the regulation of physiological responses. The present study explored whether genetic variation in the *TAS2R38* bitterness receptor is associated with body size and adiposity as well as dietary intake. For a genetic marker, *TAS2R38* rs10246939 T > C was tested since carriers with the C allele are known to have a strong intensity of bitterness while those with the T allele were less sensitive. The findings suggested that the bitterness receptor genetic variation was associated with Koreans’ dietary intake and risk for obesity, and the associations were more clearly evident in females.

Bitterness is an important sensory trait in terms of food preference [5]. Therefore, many studies have been conducted to ascertain the critical marker related to human dietary intake and bitterness perception intensity. Although inconsistencies between phenotype and genotype exist, a number of studies have provided evidence that *TAS2R38* genetic variations are a marker for individual health behavior as well as overall diet. The *TAS2R38* diplotype regulated the intake of cruciferous vegetables [7], total vegetables, and sweets [8] and the use of other bitter-tasting-compounds, i.e., tobacco and alcohol [11,29]. Furthermore, such *TAS2R38* genetic variation was evidently associated with the perception of fat [9]. Previous Korean studies reported that the *TAS2R38* diplotype was associated with tobacco use [13] and alcohol consumption [11], but did not influence the intake of cruciferous vegetables [13,14]. Current results from a nationwide cohort study also revealed that *TAS2R38* rs10246939 did not show a meaningful effect on the intake of cruciferous vegetable. Cruciferous vegetables are a major type of vegetable consumed in Koreans’ diets. However, the vegetables are generally consumed in pickled and salted dishes with a large amount of condiments, such as garlic, hot peppers, ginger, vinegar, salt, soy sauce, fish sauce, sesame oil and other artificial flavor enhancers. The strong flavor of these seasonings may mask the bitterness of cruciferous vegetables. Therefore, the bitterness receptor genetic variant did not have an influence on the vegetable intake [14]. However, the *TAS2R38* rs10246939 genetic variation was significantly associated with females’ total fruit intake. Fruits generally do not contain glucosinolates with the exception of citrus fruits. However, in terms of sensory intensity, compared with bitterness tasters, the lower bitterness perception of non-tasters may be linked to the reduced overall sensing of taste, including sweet and sour [30,31,32,33,34]. Therefore, subjects with the TT genotype, i.e., non-tasters, might consume more fruits than those with the CC (super taster) or CT genotypes (medium taster).

A number of studies including subjects of multiple ethnicities, ages and geographical regions have been conducted to investigate the associations between *TAS2R38* polymorphisms and body size and obesity, yet the findings are still controversial. *TAS2R38* polymorphisms were not associated with BMI or waist circumference in southern Italian [9] and Indian populations [21]. However, the distribution of obesity class, BMI, and body weight differed based on the *TAS2R38* diplotype in Irish girls [22] and young Japanese female adults [19]. The bitterness receptor diplotype was shown to increase the risk of obesity in a Spanish population: Individuals with the AVI/AVI diplotype were more likely to be obese, and this risk was 4.47-fold higher (95% CI: 1.05–18.94) in the subjects aged <40 years [15]. Furthermore, the diplotype was also significantly associated with the differential level of surfactant protein D and mannan binding lectin, the markers of olfactory function and immune responses [15]. A few hypotheses could be proposed for these findings. First, individuals with obesity generally possess lower olfactory and taste capacities, which may suggest impaired sensory sensitivity [35]. As described above, non-taster individuals with the TT genotype who were at risk of obesity in the current study may have lower sensitivity to overall taste, including bitterness. Therefore, non-tasters may relatively less concern about taste and controlling food intake, which may lead them to have a higher BMI. Second, *TAS2R38* genetic variations may regulate body weight via food intake modified by the endocannabinoid system [36], which controls the synthesis of N-arachidonoylethanolamide (anandamide, AEA) and 2-arachidonoylglycerol (2-AG), the derivatives of arachidonic acid [37]. Receptors for these endocannabinoids are expressed on various tissues and are known to play a role in the regulation of food intake. A previous study revealed that levels of AEA and 2-AG differed between bitterness non-tasters and super tasters [38]. Therefore, such differential bitterness intensities that may be caused by the *TAS2R38* genetic variation are associated with the control of food intake via the endocannabinoid system, which consequently influences the regulation of body weight and adiposity [36].

The regulation of food intake by *T2R38*-associated mechanisms, however, could not fully elucidate the association between body weight, obesity risk and the genetic variation. Because the *TAS2R38* rs10246939 bitterness receptor genetic variant mostly had a minimal effect on the dietary and energy intake in this Korean study cohort. The genotype was only associated with females’ fruits intake. (The *TAS2R38* rs10246939 variation predicted differences in the males’ total energy intake by the statistical model, taking into account life-styles and socioeconomic characteristics. However, this significance must be interpreted with caution. This difference was evident only in the adjusted model, which might be mainly a result of the covariates.) These findings may suggest that other extra-orally expressed T2R38 bitterness receptors may be associated with modulation of energy expenditure and adiposity metabolism independent of dietary intake control. For instance, T2R bitterness receptors are expressed in the thyroid, a critical tissue in energy metabolism [17]. Although an earlier study reported that the *TAS238* diplotype was not associated with the level of thyroid hormones [16,17], we still cannot dismiss the idea that the stimulation of agonists of T2R38 may alter thyroid function, which may lead to differences in energy metabolism, depending on the genotype. Furthermore, T2R38 expression in enteroendocrine cells varied in subjects with and without obesity. This finding suggests that genetic variation may mediate the regulation of energy balance and the intraluminal alterations of adiposity-related mechanisms in alimentary systems [39]. Additionally, the endocannabinoid system associated with bitterness sensing is known to regulate not only food intake but also energy metabolism and endocrine pancreas activity. Therefore, this system may ultimately modify the risk of obesity as well as other metabolic diseases, independent of alteration of dietary intake, or concurrently [36,40]. The interaction between taste sensitivity and weight is complicated. Subjects’ hunger and appetite and restraint of these factors could modify the intensity of taste and olfactory sensitivity [41,42]. These changes may also affect their choice of foods and body weight, interacting with other physiological metabolism, described above. The present knowledge is limited to describing the full repertoire regarding the taste sensing and adiposity-related mechanisms. Thus, the current findings could provide insights into the role of T2R bitterness receptors in the regulation of dietary behavior and physiological metabolism. More epidemiological and experimental studies are required to investigate the function of T2Rs, beyond T2R38, in human health and diseases.

Given the earlier studies, however, the findings of the present study might need more elucidation. First, consuming fruits, instead of sweets, soda and fatty foods, is known to be a healthy dietary habit in the prevention and treatment of obesity. In the current study, however, females with the TT genotype had higher fruit intake, and their weight, hip circumference and BMI were also significantly higher than those with the other genotypes. This controversial finding may be due to the characteristics of the study subjects: Middle-aged to elderly Korean rural residents. Traditional Korean diets do not contain large amounts of sweets, soda or fat but mainly consist of grains and vegetables. Individuals with the TT genotype may consume more fruits, in addition to the foods in common Korean diet. Fructose is metabolized faster than glucose [43]. High consumption of fructose would give rise to the synthesis of acetyl-CoA, promoting hepatic lipogenesis [44]. In individuals with overweight/obesity, fructose-sweetened drinks increase visceral adiposity and dyslipidemia [45]. Given the metabolic attributes of fructose, the greater intake of fruits as well as sweets for the TT homozygous subjects might contribute to their higher body weight, BMI and increased risk of obesity in addition to the T2R38-associated energy/adiposity metabolism. Second, the *TAS2R38* rs10246939 variation was significantly associated with dietary intake, body size and risk of obesity mainly in females. Although potential obesity risk modifying effect of mutation allele was present in entire cohort, when sex-stratified analysis was applied, that was less statistically significant in male subjects. The sex-specific effect of *TAS2R38* genetic variation on dietary habits and body weight was evident mainly in females [19,20,22], although few studies have reported the influence of the genetic variation in males [22,46]. Little is known that can explain such a disparity between females and males. However, from childhood, gender differences in the perception of tastes and food choices have been observed [10,22,47]. Females’ bitterness intensity could be affected by hormonal variation such as menstrual cycle; bitterness sensitivity is known to be diminished after menopause [48,49]. These may suggest that certain sex-specific physiological pathways including those involving hormones could underlie the differential association between sex and *TAS2R38* genetic variation with regard to dietary intake and adiposity [22,46]. Furthermore, females generally have more knowledge of health than males and tend to practice health-conscious behaviors [50]. Moreover, in Asian cultures, women are less likely to be smokers and drinkers, which may affect sensory intensity. Therefore, the association of the bitterness phenotype/genotype with dietary intake and body weight was more often evident in females (the *TAS2R38* genetic variation was marginally associated with females’ smoking behavior; however, it was not considered significant in the current study. This finding may be due to the small number of female smokers caused by underreporting). Lastly, although the MRCohort is a large epidemiological study cohort and sex-stratified analysis was applied, compared to the size of female group (*n* = 2229), relatively smaller male group (*n* = 1338) might lead to limited statistical power and/or the null effect of *TAS2R38* genetic variation on dietary and physiological responses.

The present study is the first to report that a *TAS2R38* genetic variation modified adiposity in Korean females, but the study may harbor a few limitations. First, as a genetic marker for bitterness sensitivity, the single locus *TAS2R38* rs10246939 was investigated. The *TAS2R38* diplotype with three variations is commonly applied for bitterness genetic studies, yet all three loci were not available from the genetic data of the MRCohort produced using an Illumina array. However, in previous Korean studies, three genetic variations were in almost complete linkage disequilibrium (*r*^2^ = 0.99), and rs10246939 was a tagger [16]. Second, the MRCohort is the one of the largest epidemiological study cohorts in Korea. This study analyzed data from approximately 3600 Koreans; however, the subjects mainly lived in rural areas and were over 40 years of age. The findings may not fully represent the entire Korean population. Moreover, dietary data were collected using FFQ. The limitations of FFQ include recall bias and accuracy issues due to the use of closed ended questions, and these issues may affect the precise estimation of the dietary intake [51]. Lastly, the adjustment for the potential effect of multiple statistical comparison was not applied. Additional studies with larger cohorts could cover the various features of Koreans should be performed to confirm the findings of the current study and improve the statistical power of the results.

## 5. Conclusions

Obesity is a multifactorial disease. Recent obesity-genetic studies have explored the genetic loci involved in multiple parts of the etiology. In line with those studies, the present study provided evidence that *TAS2R38* bitterness receptor genetic variations are a critical subject in the regulation of dietary intake as well as energy and adipose tissue metabolism. *TAS2R38* rs10246939 T allele may be associated with an increased dietary intake of fruit and a higher risk of obesity in Korean females. Further studies are necessary to examine the applicability of *TAS2R38* genetic variations as a predictive marker of the risk of obesity and metabolic diseases.

## Figures and Tables

**Table 1:** Descriptive data of the study subjects taking into account the *TAS2R38* rs10246939 genotype and sex.

	Males				Females			
	CC (*n* = 489, 36.6%)	CT (*n* = 645, 48.2%)	TT (*n* = 204, 15.3%)	*p*-Value	CC (*n* = 744, 33.4%)	CT (*n* = 1081, 48.5%)	TT (*n* = 404, 18.1%)	*p*-Value
Age	60.94 (10.16)	61.6 (9.99)	61.51(8.66)	0.460	58.6 (9.84)	59.23 (10.09)	58 (10.01)	0.099
Tobacco smoking								
Never	125 (25.56)	139 (21.55)	35 (14.16)	0.064	712 (95.7)	1022 (94.54)	381 (94.31)	0.041
Past	183 (37.42)	235 (36.43)	89 (43.63)		11 (1.48)	23 (2.13)	2 (0.5)	
Current	181 (37.01)	271 (42.02)	79 (38.73)		20 (2.69)	36 (3.33)	21 (5.2)	
Missing			1 (0.49)		1 (0.13)			
Alcohol drinking								
Never	130 (26.58)	162 (24.12)	46 (22.55)	0.879	457 (61.42)	715 (66.14)	263 (65.1)	0.343
Past	51 (10.43)	69 (10.7)	22 (10.78)		20 (2.69)	23 (2.13)	10 (2.48)	
Current	308 (62.99)	41 (64.19)	135 (66.18)		266 (35.75)	342 (31.64)	131 (32.43)	
Missing			1 (0.49)		1 (0.13)	1 (0.09)		
Education level								
Elementary	347 (50.51)	347 (53.8)	102 (50.0)	0.293	526 (70.7)	738 (68.27)	285 (70.54)	0.186
High school	192 (39.26)	241 (37.36)	75 (36.76)		179 (24.06)	302 (27.94)	99 (24.5)	
College/more	47 (9.61)	54 (8.37)	27 (13.24)		37 (4.97)	38 (3.52)	20 (4.95)	
Missing	3 (0.61)	3 (0.61)	-		2 (0.27)	3 (0.28)	-	
Regular exercise								
No	353 (72.19)	484 (75.04)	150 (73.53)	0.555	534 (71.77)	795 (73.54)	303 (75.0)	0.465
Yes	136 (27.81)	161 (24.96)	54 (26.47)		210 (28.23)	285 (26.36)	101 (25.0)	
Missing	-	-	-		-	1 (0.09)	-	

Values denote the means ± standard deviations for age; otherwise, the data present numbers of subjects and percentages in parentheses. The *p*-values are from the chi-squared tests among three genotypes except those for age. The *p*-values for age are from the generalized linear models among the three genotypes.

**Table 2:** Mean intake of selected food groups and alcohol taking into account the *TAS2R38* rs10246939 genotype and sex.

	CC	CT	TT	*p*-Value *	*p*-Value **
Males	*n* = 489	*n* = 645	*n* = 204		
Vegetables (g/day)	252.9 (6.87)	250.4 (5.75)	242.6 (10.7)	0.321	0.317
Cruciferous (g/day)	188.8 (5.88)	186.1 (4.92)	182.1 (9.25)	0.553	0.600
Dark green (g/day)	22.8 (1.25)	22.1 (0.81)	21.5 (1.25)	0.919	0.957
Fruits (g/day)	116.3 (4.01)	120.0 (4.28)	123.9 (7.29)	0.870	0.825
Citrus (g/day)	22.9 (1.22)	22.7 (1.18)	21.2 (2.0)	0.227	0.239
Fiber (g/day)	4.90 (0.99)	4.79 (0.07)	4.66 (0.12)	0.343	0.518
Sweets (g/day)	28.0 (1.93)	27.5 (1.53)	26.3 (2.93)	0.336	0.342
Alcohol (g/day)	35.9 (3.07)	33.3 (2.17)	37.9 (4.33)	0.652	0.628
Total energy (kcal/day)	1699.7 (23.9)	1761.9 (20.7)	1722.2 (37.5)	0.135	0.024
Percent of energy from					
Carbohydrate	75.9 (0.28)	75.7 (0.25)	75.5 (0.42)	0.671	0.736
Protein	12.3 (0.09)	12.4 (0.09)	12.4 (0.17)	0.444	0.465
Fat	11.7 (0.21)	11.9 (0.19)	11.9 (0.31)	0.538	0.657
Females	*n* = 744	*n* = 1081	*n* = 404		
Vegetables (g/day)	249.5 (5.69)	247.1 (4.70)	242.3 (6.74)	0.978	0.809
Cruciferous (g/day)	174.1 (4.79)	168.4 (3.78)	166.9 (5.56)	0.829	0.987
Dark green (g/day)	24.8 (0.85)	25.1 (0.81)	24.9 (1.28)	0.796	0.980
Fruits (g/day)	163.6 (5.44)	169.8 (4.48)	184.3 (7.46)	0.031	0.025
Citrus (g/day)	33.8 (1.74)	34.6 (1.38)	37.1 (2.18)	0.286	0.280
Fiber (g/day)	5.13 (0.08)	5.12 (0.06)	5.10 (0.10)	0.294	0.990
Sweets (g/day)	24.1 (1.74)	26.5 (1.34)	29.5 (2.74)	0.282	0.214
Alcohol (g/day)	6.92 (1.38)	7.11 (0.74)	6.41 (1.03)	0.548	0.566
Total energy (kcal/day)	1548.7 (18.1)	1494.6 (13.2)	1534.3 (23.2)	0.089	0.161
Percent of energy from					
Carbohydrate	77.8 (0.23)	77.4 (0.20)	77.5 (0.35)	0.819	0.741
Protein	12.0 (0.08)	12.1 (0.07)	11.9 (0.10)	0.733	0.710
Fat	10.1 (0.18)	10.5 (0.15)	10.5 (0.27)	0.275	0.326

Numbers in brackets are standard errors. * *p*-values are from the crude generalized linear model among the three genotypes. ** *p*-values are from the generalized linear model adjusted for age, education level, alcohol consumption and tobacco use, regular exercise and total energy intake.

**Table 3:** Mean body size markers taking into account the *TAS2R38* rs10246939 genotype and sex.

	CC	CT	TT	*p*-Value *	*p*-Value **
Males	*n* = 489	*n* = 645	*n* = 204		
Height (cm)	165.2 (0.30)	165.2 (0.25)	164.9 (0.46)	0.783	0.845
Weight (kg)	64.1 (0.46)	64.2 (0.39)	64.2 (0.70)	0.926	0.575
Waist (cm)	84.1 (0.38)	84.4 (0.33)	84.3 (0.59)	0.874	0.829
Hip (cm)	92.6 (0.30)	92.6 (0.25)	92.3 (0.45)	0.748	0.914
Body mass index (kg/m^2^)	23.4 (0.13)	23.5 (0.12)	23.5 (0.21)	0.760	0.364
Ideal body weight (kg)	58.7 (0.27)	58.7 (0.22)	58.4 (0.41)	0.788	0.836
Females	*n* = 744	*n* = 1081	*n* = 404		
Height (cm)	153.0 (0.21)	152.9 (0.19)	152.9 (0.29)	0.880	0.524
Weight (kg)	55.9 (0.30)	56.0 (0.26)	57.3 (0.43)	0.019	0.046
Waist (cm)	81.8 (0.33)	82.2 (0.27)	82.6 (0.46)	0.389	0.218
Hip (cm)	92.4 (0.24)	92.6 (0.20)	93.4 (0.33)	0.048	0.045
Body mass index (kg/m^2^)	23.8 (0.11)	23.9 (0.10)	24.5 (0.16)	0.002	0.003
Ideal body weight (kg)	47.7 (0.19)	47.6 (0.17)	47.6 (0.26)	0.833	0.542

Numbers in brackets are standard errors. * *p*-values are from the crude generalized linear model among the three genotypes. ** *p*-values are from the generalized linear model adjusted for age, education level, alcohol consumption and tobacco use, regular exercise and total energy and fruit intake.

**Table 4:** Distribution of underweight, normal, overweight and obese subjects defined by their body mass index taking into account the *TAS2R38* rs10246939 genotype and sex.

	Underweight	Normal	Overweight	Obesity	*p*-Value
Males					
CC	23 (41.82)	205 (37.55)	107 (36.15)	151 (35.53)	0.803
CT	25 (45.45)	265 (48.53)	141 (47.64)	203 (47.76)	
TT	7 (12.73)	76 (13.92)	48 (16.22)	71 (16.71)	
Females					
CC	11 (17.46)	304 (37.53)	194 (34.28)	229 (29.93)	0.005
CT	41 (65.08)	378 (46.67)	279 (49.29)	368 (48.10)	
TT	11 (17.46)	128 (15.8)	93 (16.43)	168 (21.96)	

Values denote the number of subjects. Numbers in brackets indicate the percent of subjects. Classes of obesity were defined based on a body mass index (BMI, kg/m^2^) < 18.5, 18.5 ≤ BMI < 23, 23 ≤ BMI < 24.9, and BMI ≥ 25. *p*-values are from the chi-squared tests. Underweight subjects were excluded from the tests due to their rarity.

**Table 5:** Association between the *TAS2R38* rs10246939 genotype and the risk of being overweight and obese in males and females.

	Overweight			Obesity		
	OR (95% CI)	OR (95% CI)	*p*-Value *	OR (95% CI)	OR (95% CI)	*p*-Value *
	Model I	Model II		Model I	Model II	
Males						
CC	reference	reference		reference	reference	
CT	1.02 (0.74–1.39)	1.01 (0.73–1.39)	0.961	1.04 (0.78–1.37)	1.09 (0.81–1.47)	0.541
TT	1.21 (0.78–1.86)	1.23 (0.78–1.95)	0.362	1.26 (0.86–1.86)	1.45 (0.95–2.22)	0.081
Females						
CC	reference	reference		reference	reference	
CT	1.15 (0.91–1.46)	1.24 (0.97–1.58)	0.084	1.29 (1.03–1.61)	1.33 (1.06–1.67)	0.014
TT	1.13 (0.82–1.57)	1.19 (0.85–1.66)	0.309	1.74 (1.31–2.32)	1.75 (1.31–2.36)	0.0002
All						
CC	reference	reference		reference	reference	
CT	1.11 (0.92–1.33)	1.15 (0.95–1.39)	0.151	1.19 (0.99–1.42)	1.23 (1.02–1.47)	0.027
TT	1.17 (0.90–1.51)	1.21 (0.93–1.57)	0.163	1.57 (1.25–1.97)	1.61 (1.27–2.04)	<0.0001

OR, odds ratio; 95% CI, 95% confidence interval. OR values were estimated based on a comparison with normal subjects. Underweight individuals were excluded from the model due to their rarity. ORs were estimated from the additive models (CC versus CT and CC versus TT). Model I: crude model. Model II: adjusted for age, education level, alcohol consumption, tobacco use, regular exercise and total energy and fruit intake. * *p*-value for the OR of the adjusted model II.

## Supplementary Materials

The following are available online at https://www.mdpi.com/2072-6643/11/9/1973/s1, Table S1: The food groups analyzed in the current study.
