# Supplementary material for: Variation in the TAS2R38 Bitterness Receptor Gene Was Associated with Food Consumption and Obesity Risk in Koreans
Source: Nutrients. 2019 Aug 21;11(9):1973. doi: 10.3390/nu11091973 (PMC6770895; doi:10.3390/nu11091973)
Supplement: Supplementary file 1 [file nutrients-11-01973-s001.pdf]

Supplementary Table S1. The food groups analyzed in the current study

Group	Food items
Vegetables	all vegetables
Cruciferous vegetables	napa cabbage, shepherd's purse, white radish and other cruciferous vegetables
Dark green vegetables	chives, crown daisy, doellingeria scaber leaf, green pepper, green pepper leaf, lettuce, perilla leaf, pimpinella brachycarpa leaf, shepherd's purse, spinach, water parsley and other dark green vegetables
Fruits	all fruits and fruits products
Citrus fruits	mandarin, orange and their products
Sweets	cake, candy, chocolate, chocolate pie, cookie, cracker, ice cream, soda and sugar for tea
